# On Some Fuzzy Filters in Pseudo-*BCI* Algebras

**DOI:** 10.1155/2014/718972

**Published:** 2014-04-08

**Authors:** Xiaohong Zhang

**Affiliations:** College of Arts and Sciences, Shanghai Maritime University, Shanghai 201306, China

## Abstract

Some new properties of fuzzy associative filters (also known as fuzzy associative pseudo-filters), fuzzy *p*-filter (also known as fuzzy pseudo-*p*-filters), and fuzzy *a*-filter (also known as fuzzy pseudo-*a*-filters) in pseudo-*BCI* algebras are investigated. By these properties, the following important results are proved: (1) a fuzzy filter (also known as fuzzy pseudo-filters) of a pseudo-*BCI* algebra is a fuzzy associative filter if and only if it is a fuzzy *a*-filter; (2) a filter (also known as pseudo-filter) of a pseudo-*BCI* algebra is associative if and only if it is an *a*-filter (also call it pseudo-*a* filter); (3) a fuzzy filter of a pseudo-*BCI* algebra is fuzzy *a*-filter if and only if it is both a fuzzy *p*-filter and a fuzzy *q*-filter.

## 1. Introduction and Preliminaries

In the field of artificial intelligence research, nonclassical logics (fuzzy logic, epistemic logic, nonmonotonic logic, default logic, etc.) are extensively used (see [1]). In this paper we discuss a kind of logic algebra system, that is, pseudo-*BCI* algebra, which originated from *BCI*-logic; it is a kind of nonclassical logic and inspired by the calculus of combinators [2]. Newly, in [3], we show that pseudo-*BCI* algebra plays an important role in weakly integral residuated algebraic structure, which is in close connection with various fuzzy logic formal systems [4, 5].

In 1966, Iséki [2] introduced the concept of *BCI*-algebra as an algebraic counterpart of the *BCI*-logic. Since then, the ideal theory of *BCI*-algebras gets in-depth research and development. In 2008, as a generalization of *BCI*-algebra, Dudek and Jun [6] introduced the notion of pseudo-*BCI* algebra which is also generalization of pseudo-*BCK* algebra introduced by Georgescu and Iorgulescu in [7]. We investigated some classes of pseudo-*BCI* algebras in [8]. Recently, the pseudoideal theory of pseudo-*BCI* algebras has been studied: the notion of pseudo-*BCI* ideal (or pseudoideal) of pseudo-*BCI* algebra is introduced in [9]; some special pseudo-*BCI* ideals are discussed in [10], for example, associative pseudoideal and pseudo-*a* ideal, which are generalization of associative ideal of *BCI*-algebra (it is introduced by X.H. Zhang and R.G. Ling).

The notion of fuzzy sets has been applied to many algebraic systems (see [4, 11, 12]); naturally, it has been applied to pseudo-*BCK*/*BCI* algebra; for example, fuzzy pseudoideals have been investigated in [13–15]. As continuums of the above works, we further study fuzzy associative pseudoideal and fuzzy pseudo-*a* ideal in pseudo-*BCI* algebras.

Note that the notion of pseudo-*BCI* algebra in this paper is indeed dual form of original definition in [6]; accordingly, the notion of pseudo-filter (or pseudo-*BCI* filter) is the dual form of pseudoideal (or pseudo-*BCI* ideal) in [9]. Moreover, for short, the notion of pseudo-filter (or pseudo-*BCI* filter) is simply called “filter” in this paper.

At first, we recall some basic concepts and properties of pseudo-*BCI* algebras.

**Definition (see [6])**

A pseudo-*BCI* algebra is a structure (*X*; ≤, →, ⇝, 1), where “≤” is a binary relation on *X*, “→” and “⇝” are binary operations on *X*, and “1” is an element of *X*, verifying the axioms: for all *x*, *y*, *z* ∈ *X*,

1. *y* → *z* ≤ (*z* → *x*)⇝(*y* → *x*), *y*⇝*z* ≤ (*z*⇝*x*)→(*y*⇝*x*);
2. *x* ≤ (*x* → *y*)⇝*y*, *x* ≤ (*x*⇝*y*) → *y*;
3. *x* ≤ *x*;
4. *x* ≤ *y*, *y* ≤ *x*⇒*x* = *y*;
5. *x* ≤ *y*⇔*x* → *y* = 1⇔*x*⇝*y* = 1.

If (*X*; ≤, →, ⇝, 1) is a pseudo-*BCI* algebra satisfying *x* → *y* = *x*⇝*y* for all *x*, *y* ∈ *X*, then (*X*; ≤, →, ⇝, 1) is a *BCI*-algebra.

**Proposition 2 (see [6, 9, 10])**

Let (*X*; ≤, →, ⇝, 1) be a pseudo-*BCI* algebra; then *X* satisfy the following properties (∀*x*, *y*, *z* ∈ *X*):

1. 1 ≤ *x*⇒*x* = 1*;*
2. *x* ≤ *y*⇒*y* → *z* ≤ *x* → *z*, *y*⇝*z* ≤ *x*⇝*z;*
3. *x* ≤ *y*, *y* ≤ *z*⇒*x* ≤ *z;*
4. *x*⇝(*y* → *z*) = *y* → (*x*⇝*z*)*;*
5. *x* ≤ *y* → *z*⇒*y* ≤ *x*⇝*z;*
6. *x* → *y* ≤ (*z* → *x*)→(*z* → *y*), *x*⇝*y* ≤ (*z*⇝*x*)⇝(*z*⇝*y*)*;*
7. *x* ≤ *y*⇒*z* → *x* ≤ *z* → *y*, *z*⇝*x* ≤ *z*⇝*y;*
8. 1 → *x* = *x*, 1⇝*x* = *x;*
9. ((*y* → *x*)⇝*x*) → *x* = *y* → *x*, ((*y*⇝*x*) → *x*)⇝*x* = *y*⇝*x;*
10. *x* → *y* ≤ (*y* → *x*)⇝1, *x*⇝*y* ≤ (*y*⇝*x*) → 1*;*
11. (*x* → *y*) → 1 = (*x* → 1)⇝(*y*⇝1), (*x*⇝*y*)⇝1 = (*x*⇝1)→(*y* → 1)*;*
12. *x* → 1 = *x*⇝1.

**Definition 3 (see [9])**

A nonempty subset *F* of a pseudo-*BCI* algebra *X* is called a pseudo-*BCI* filter (briefly, filter) of *X* if it satisfies

(F1) 1 ∈ *F*;
(F2) *x* ∈ *F*, *x* → *y* ∈ *F*⇒*y* ∈ *F*;
(F3) *x* ∈ *F*, *x*⇝*y* ∈ *F*⇒*y* ∈ *F*.

**Definition 4 (see [10])**

A nonempty subset *F* of a pseudo-*BCI* algebra *X* is called an associative pseudo-*BCI* filter (briefly, associative filter) of *X* if it satisfies

1. 1 ∈ *F*;
2. *x* → (*y*⇝*z*) ∈ *F* and *x* → *y* ∈ *F*⇒*z* ∈ *F*;
3. *x*⇝(*y* → *z*) ∈ *F* and *x*⇝*y* ∈ *F*⇒*z* ∈ *F*.

**Definition 5 (see [10])**

A nonempty subset *F* of a pseudo-*BCI* algebra *X* is called a pseudo-*a*-filter (briefly, *a*-filter) of *X* if it satisfies

1. 1 ∈ *F*;
2. (*x* → 1)⇝(*y* → *z*) ∈ *F* and *y* ∈ *F*⇒*z*⇝*x* ∈ *F*;
3. (*x*⇝1)→(*y*⇝*z*) ∈ *F* and *y* ∈ *F*⇒*z* → *x* ∈ *F*.

**Definition 6 (see [13–15])**

A fuzzy set *μ* : *X* → [0,1] is called a fuzzy pseudofilter (briefly, fuzzy filter) of pseudo-*BCI* algebra *X* if it satisfies

(FF1) *μ*(1) ≥ *μ*(*x*), ∀*x* ∈ *X*;
(FF2) *μ*(*y*) ≥ min⁡{*μ*(*x* → *y*), *μ*(*x*)}, ∀*x*, *y* ∈ *X*;
(FF3) *μ*(*y*) ≥ min⁡{*μ*(*x*⇝*y*), *μ*(*x*)}, ∀*x*, *y* ∈ *X*.

**Proposition 7**

Let *μ* be a fuzzy filter of a pseudo-*BCI* algebra *X*. If *x* ≤ *y*, then *μ*(*x*) ≤ *μ*(*y*), where *x*, *y* ∈ *X*.

As a consequence of the so-called* Transfer Principle for Fuzzy Sets* in [11], we have the following.

**Theorem (see [11, 15])**

Let *X* be a pseudo-*BCI* algebra. Then a fuzzy set *μ* : *X* → [0,1] is a fuzzy filter of *X* if and only if the level set *μ*_*t*_ = {*x* ∈ *X* | *μ*(*x*) ≥ *t*} is filter of *X* for all *t* ∈ *Im*⁡(*μ*).

**Theorem 9 (see [15])**

Let *X* be a pseudo-*BCI* algebra. Then a fuzzy set *μ* : *X* → [0,1] is a fuzzy filter of *X* if and only if it satisfies

1. for all *x*, *y*, *z* ∈ *X*, *x* ≤ *y* → *z*⇒*μ*(*z*) ≥ min⁡{*μ*(*x*), *μ*(*y*)};
2. for all *x*, *y*, *z* ∈ *X*, *x* ≤ *y*⇝*z*⇒*μ*(*z*) ≥ min⁡{*μ*(*x*), *μ*(*y*)}.

**Definition 10 (see [13, 15])**

A fuzzy set *μ* : *X* → [0,1] is called a fuzzy *p*-filter of a pseudo-*BCI* algebra *X* if it satisfies (FF1) and

(FPF1) for all *x*, *y*, *z* ∈ *X*, *μ*(*z*) ≥ min⁡{*μ*((*x* → *y*)⇝(*x* → *z*)), *μ*(*y*)};
(FPF2) for all *x*, *y*, *z* ∈ *X*, *μ*(*z*) ≥ min⁡{*μ*((*x*⇝*y*)→(*x*⇝*z*)), *μ*(*y*)}.

**Definition 11 (see [13, 15])**

A fuzzy set *μ* : *X* → [0,1] is called a fuzzy *a*-filter of a pseudo-*BCI* algebra *X* if it satisfies (FF1) and

(FaF1) for all *x*, *y*, *z* ∈ *X*, *μ*(*z* → *x*) ≥ min⁡{*μ*((*x*⇝1)→(*y*⇝*z*)), *μ*(*y*)};
(FaF2) for all *x*, *y*, *z* ∈ *X*, *μ*(*z*⇝*x*) ≥ min⁡{*μ*((*x* → 1)⇝(*y* → *z*)), *μ*(*y*)}.

**Definition 12 (see [15])**

A fuzzy set *μ* : *X* → [0,1] is called a fuzzy associative filter of a pseudo-*BCI* algebra *X* if it satisfies (FF1) and

(FAF1) for all *x*, *y*, *z* ∈ *X*, *μ*(*z*) ≥ min⁡{*μ*(*x* → (*y*⇝*z*)), *μ*(*x* → *y*)};
(FAF2) for all *x*, *y*, *z* ∈ *X*, *μ*(*z*) ≥ min⁡{*μ*(*x*⇝(*y* → *z*)), *μ*(*x*⇝*y*)}.

**Definition 13 (see [13, 15])**

A fuzzy set *μ* : *X* → [0,1] is called a fuzzy *q*-filter of a pseudo-*BCI* algebra *X* if it satisfies (FF1) and

(FqF1) for all *x*, *y*, *z* ∈ *X*, *μ*(*x* → *z*) ≥ min⁡{*μ*((*x*⇝*y*) → *z*), *μ*(*y*)};
(FqF2) for all *x*, *y*, *z* ∈ *X*, *μ*(*x*⇝*z*) ≥ min⁡{*μ*((*x* → *y*)⇝*z*), *μ*(*y*)}.

## 2. New Properties of Fuzzy *a*-Filters and Fuzzy Associative Filters

**Lemma 14 (see [15])**

Let *μ* be a fuzzy *a*-filter of a pseudo-*BCI* algebra *X*. Then *μ* satisfies

(1) μ(x⟶y)≥μ((y⇝1)⟶x),μ(x⇝y)≥μ((y⟶1)⇝x),         ∀x,y∈X.

**Lemma 15 (see [15])**

Let *μ* be a fuzzy *a*-filter of a pseudo-*BCI* algebra *X*. Then *μ* satisfies

(2) $$\begin{array}{c} \mu \left(x\right)=\mu \left(x⟶1\right)=\mu \left(x⇝1\right),\;\forall x\in X. \end{array}$$

**Theorem 16**

Let *μ* be a fuzzy *a*-filter of a pseudo-*BCI* algebra *X*. Then the following statements hold for all *x*, *y*, *z* ∈ *X*:

1. for all *x*, *y* ∈ *X*, *μ*(*x*) ≥ min⁡{*μ*(*y*), *μ*(*x* → *y*)}, *μ*(*x*) ≥ min⁡{*μ*(*y*), *μ*(*x*⇝*y*)};
2. for all *x*, *y* ∈ *X*, *μ*(*x* → *y*) = *μ*(*x*⇝*y*);
3. for all *x* ∈ *X*, *μ*((*x* → 1) → *x*) = *μ*((*x*⇝1)⇝*x*) = *μ*(1);
4. for all *x*, *y*, *z* ∈ *X*, *μ*(*x* → *z*) ≥ min⁡{*μ*((*x*⇝*y*) → *z*), *μ*(*y*)};
5. for all *x*, *y*, *z* ∈ *X*, *μ*(*x*⇝*z*) ≥ min⁡{*μ*((*x* → *y*)⇝*z*), *μ*(*y*)};
6. for all *x* ∈ *X*, *μ*(*x* → (*x*⇝1)) = *μ*(*x*⇝(*x* → 1)) = *μ*(1);
7. for all *x*, *y*, *z* ∈ *X*, *μ*((*x* → *y*) → *y*) ≥ *μ*(*x*), *μ*((*x*⇝*y*)⇝*y*) ≥ *μ*(*x*).

**Proof**

(1) For any *x*, *y* ∈ *X*, by Proposition 2(6), we have *y* → 1 ≤ (*x* → *y*)→(*x* → 1). From this, applying Proposition 7, we get

(3) $$\begin{array}{c} \mu \left(y⟶1\right)\leq \mu \left(\left(x⟶y\right)⟶\left(x⟶1\right)\right). \end{array}$$

Using Definition 6(FF2), we have

(4) μ(x⟶1) ≥min⁡{μ((x⟶y)⟶(x⟶1)),μ(x⟶y)}.

Thus,

(5) μ(x⟶1) ≥min⁡⁡{μ((x⟶y)⟶(x⟶1)),μ(x⟶y)} ≥min⁡{μ(y⟶1),μ(x⟶y)}.  

By Lemma 15, *μ*(*x* → 1) = *μ*(*x*) and *μ*(*y* → 1) = *μ*(*y*). Therefore, *μ*(*x*) ≥ min⁡{*μ*(*y*), *μ*(*x* → *y*)}.

Similarly, we have *μ*(*x*) ≥ min⁡{*μ*(*y*), *μ*(*x*⇝*y*)}.

(2) For any *x*, *y* ∈ *X*, by Proposition 2(11) and (12), we have (*x* → *y*) → 1 = (*x* → 1)⇝(*y* → 1). By Lemma 14, it follows that

(6) μ((x⟶y)⟶1)=μ((x⟶1)⇝(y⟶1))≤μ((y⟶1)⇝x)≤μ(x⇝y).

From this, applying Lemma 15, we get *μ*(*x* → *y*) ≤ *μ*(*x*⇝*y*). Similarly, we can get *μ*(*x*⇝*y*) ≤ *μ*(*x* → *y*). By Definition 1(4), *μ*(*x* → *y*) = *μ*(*x*⇝*y*).

(3) For any *x* ∈ *X*, applying Proposition 2(12) and Lemma 14, we have

(7) μ((x⟶1)⟶x)=μ((x⇝1)⟶x)≥μ((x⇝1)⟶(x⇝1))=μ(1).

By Definition 6(FF1), it follows that *μ*((*x* → 1) → *x*) = *μ*(1).

Similarly, we have *μ*((*x*⇝1)⇝*x*) = *μ*(1).

(4) For any *x*, *y* ∈ *X*, by Proposition 2(6) and Definition 1(1), we have

(8) y⇝1≤(x⇝y)⇝(x⇝1)≤((x⇝1)⇝x)⟶((x⇝y)⇝x).

From this, by Theorem 8, we get

(9) $$\begin{array}{c} \mu \left(\left(x⇝y\right)⇝x\right)\geq \min\left\{\mu \left(y⇝1\right),\mu \left(\left(x⇝1\right)⇝x\right)\right\}. \end{array}$$

Applying (3), Lemma 15, and Definition 6(FF1), *μ*((*x*⇝*y*)⇝*x*) ≥ *μ*(*y*). From this and (2), we get *μ*((*x*⇝*y*) → *x*) ≥ *μ*(*y*).

On the other hand, by Definition 1(1), (*x*⇝*y*) → *x* ≤ (*x* → *z*)⇝((*x*⇝*y*) → *z*). Using Proposition 7 and the above result, we have

(10) μ(y)≤μ((x⇝y)⟶x)≤μ((x⟶z)⇝((x⇝y)⟶z)).

Moreover, by (1), we have

(11) μ(x⟶z)≥min⁡{μ((x⇝y)⟶z),        μ((x⟶z)⇝((x⇝y)⟶z))}.

Therefore,

(12) μ(x⟶z)≥min⁡⁡{μ((x⇝y)⟶z),   μ((x⟶z)⇝((x⇝y)⟶z))}≥min⁡{μ((x⇝y)⟶z),μ(y)}.

This means that (4) holds.

(5) The proof is similar to (4).

(6) For any *x* ∈ *X*, by (4), we have

(13) μ(x⟶(x⇝1))≥min⁡⁡{μ((x⇝1)⟶(x⇝1)),μ(1)}=min⁡{μ(1),μ(1)}=μ(1).

It follows that *μ*(*x* → (*x*⇝1)) = *μ*(1). Similarly, *μ*(*x*⇝(*x* → 1)) = *μ*(1).

(7) For any *x* ∈ *X*, by Definition 1(3), we have *x* ≤ (*x* → *y*)⇝*y*. From this, (2), and Proposition 7, we get

(14) $$\begin{array}{c} \mu \left(x\right)\leq \mu \left(\left(x⟶y\right)⇝y\right)=\mu \left(\left(x⟶y\right)⟶y\right). \end{array}$$

Similarly, we have *μ*((*x*⇝*y*)⇝*y*) ≥ *μ*(*x*).

**Lemma 17**

Let *μ* be a fuzzy associative filter of a pseudo-*BCI* algebra *X*. Then *μ* satisfies

(15) μ(y)≥μ(x⟶(x⇝y)),μ(y)≥μ(x⇝(x⟶y)),       ∀x,y∈X.

**Proof**

It is easily proved by Definition 12; the proof is omitted.

**Theorem**

Let *μ* be a fuzzy associative filter of a pseudo-*BCI* algebra *X*. Then the following statements hold:

1. for all *x* ∈ *X*, *μ*(*x* → (*x*⇝1)) = *μ*(*x*⇝(*x* → 1)) = *μ*(1);
2. for all *x*, *y* ∈ *X*, *μ*(*y*) ≥ *μ*((*x* → 1)⇝(*x* → *y*));
3. for all *x*, *y* ∈ *X*, *μ*(*y*) ≥ *μ*((*x*⇝1)→(*x*⇝*y*));
4. for all *x*, *y* ∈ *X*, *μ*(*x* → *y*) = *μ*(*x*⇝*y*);
5. for all *x*, *y* ∈ *X*, *μ*(*x* → (*x* → *y*)) ≥ *μ*(*y*), *μ*(*x*⇝(*x*⇝*y*)) ≥ *μ*(*y*);
6. for all *x* ∈ *X*, *μ*(*x*) ≥ *μ*((*x* → 1) → 1)), *μ*(*x*) ≥ *μ*((*x*⇝1)⇝1));
7. for all *x*, *y* ∈ *X*, *μ*(*x* → (*y* → 1)) ≥ *μ*(*x* → *y*), *μ*(*x*⇝(*y*⇝1)) ≥ *μ*(*x*⇝*y*);
8. for all *x*, *y* ∈ *X*, *μ*(*y* → *x*)) ≥ *μ*(*x* → *y*), *μ*(*y*⇝*x*)) ≥ *μ*(*x*⇝*y*);
9. for all *x* ∈ *X*, *μ*((*x*⇝1) → *x*) = *μ*((*x* → 1)⇝*x*) = *μ*(1);
10. for all *x*, *y* ∈ *X*, *μ*((*x* → *y*) → *y*) ≥ *μ*(*x*), *μ*((*x*⇝*y*)⇝*y*) ≥ *μ*(*x*);
11. for all *x* ∈ *X*, *μ*(*x*) = *μ*(*x* → 1) = *μ*(*x*⇝1);
12. for all *x*, *y* ∈ *X*, *μ*(*x*) ≥ min⁡{*μ*(*y*), *μ*(*x* → *y*)}, *μ*(*x*) ≥ min⁡{*μ*(*y*), *μ*(*x*⇝*y*)}.

**Proof**

(1) For any *x* ∈ *X* (by Definition 1 and Proposition 2),

(16) (x⇝1)⇝((x⇝1)⟶(x⟶(x⇝1))) =(x⇝1)⇝(x⟶((x⇝1)⟶(x⇝1))) =(x⇝1)⇝(x⟶1) =(x⟶1)⇝(x⟶1)=1.

From this and Lemma 17, we have

(17) μ(x⟶(x⇝1)) ≥μ((x⇝1)⇝((x⇝1)⟶(x⟶(x⇝1))))=μ(1).

This means that *μ*(*x* → (*x*⇝1)) = *μ*(1). Similarly, *μ*(*x*⇝(*x* → 1)) = *μ*(1).

(2) For any *x*, *y* ∈ *X*, by Definition 1(1), we have

(18) x⇝(x⟶1) ≤((x⟶1)⇝(x⟶y))⟶(x⇝(x⟶y)).

Applying Theorem 9(1), we get

(19) μ(x⇝(x⟶y)) ≥min⁡{μ(x⇝(x⟶1)),μ((x⟶1)⇝(x⟶y))}.

By (1) and Lemma 17, *μ*(*x*⇝(*x* → 1)) = *μ*(1), *μ*(*y*) ≥ *μ*(*x*⇝(*x* → *y*)). Therefore,

(20) μ(y)≥μ(x⇝(x⟶y))  ≥min⁡{μ(1),μ((x⟶1)⇝(x⟶y))}  =μ((x⟶1)⇝(x⟶y)).

(3) It is similar to (2).

(4) For any *x*, *y* ∈ *X*, by (2), we have *μ*(*x*⇝*y*) ≥ *μ*((*y* → 1)⇝(*y* → (*x*⇝*y*))). On the other hand, applying Definition 1 and Proposition 2,

(21) (y⟶1)⇝(y⟶(x⇝y)) =(y⟶1)⇝(x⇝(y⟶y)) =(y⟶1)⇝(x⇝1) =(y⟶1)⇝(x⟶1) ≥x⟶y.

From this and Proposition 7, *μ*((*y* → 1)⇝(*y* → (*x*⇝*y*))) ≥ *μ*(*x* → *y*). Thus, *μ*(*x*⇝*y*) ≥ *μ*(*x* → *y*). Similarly, we can get *μ*(*x* → *y*) ≥ *μ*(*x*⇝*y*). Therefore, *μ*(*x* → *y*) = *μ*(*x*⇝*y*).

(5) For any *x*, *y* ∈ *X*, since (by Proposition 2)

(22) y⇝(x⟶(x⟶y))=x⟶(y⇝(x⟶y))=x⟶(x⟶(y⇝y))=x⟶(x⟶1)=x⟶(x⇝1),

then *μ*(*y*⇝(*x* → (*x* → *y*))) = *μ*(*x* → (*x*⇝1)). By (1), *μ*(*x* → (*x*⇝1)) = *μ*(1); hence, *μ*(*y*⇝(*x* → (*x* → *y*))) = *μ*(1). Moreover, by Definition 6(FF3), we have

(23) μ(x⟶(x⟶y)) ≥min⁡{μ(y⇝(x⟶(x⟶y))),μ(y)}.

Thus,

(24) μ(x⟶(x⟶y)) ≥min⁡⁡{μ(y⇝(x⟶(x⟶y))),μ(y)} =min⁡{μ(1),μ(y)}=μ(y).

Similarly, *μ*(*x*⇝(*x*⇝*y*)) ≥ *μ*(*y*).

(6) By (2) and (3), we can get (6).

(7) For any *x*, *y* ∈ *X*, by Definition 1, we have *y* → (*y* → 1))≤(*x* → *y*)→(*x* → (*y* → 1)).

Applying (1) and Theorem 9, we get

(25) μ(x⟶(y⟶1)) ≥min⁡{μ(y⟶(y⟶1)),μ(x⟶y)} =min⁡{μ(1),μ(x⟶y)}=μ(x⟶y).

Similarly, *μ*(*x*⇝(*y*⇝1)) ≥ *μ*(*x*⇝*y*).

(8) For any *x*, *y* ∈ *X*, by Lemma 17, *μ*(*y* → *x*) ≥ *μ*(*x*⇝(*x* → (*y* → *x*)))  . And, using Proposition 2(4), we have

(26) x⇝(x⟶(y⟶x)) =x⟶(x⇝(y⟶x))=x⟶(y⟶(x⇝x)) =x⟶(y⟶1).

Hence, *μ*(*y* → *x*) ≥ *μ*(*x* → (*y* → 1)). From this and (7), we get

(27) $$\begin{array}{c} \mu \left(y⟶x\right)\geq \mu \left(x⟶\left(y⟶1\right)\right)\geq \mu \left(x⟶y\right). \end{array}$$

Similarly, *μ*(*y*⇝*x*) ≥ *μ*(*x*⇝*y*).

(9) By (1) and (8), we can get (8).

(10) It is similar to the proof of Theorem 16(7).

(11) For any *x* ∈ *X*, by Lemma 17, *μ*(*x*) ≥ *μ*(*x* → (*x*⇝*x*)) = *μ*(*x* → 1). On the other hand, using (8), *μ*(*x* → 1) ≥ *μ*(1 → *x*) = *μ*(*x*). Hence, *μ*(*x*) = *μ*(*x* → 1) = *μ*(*x*⇝1).

(12) It is similar to the proof of Theorem 16(1).

## 3. Some Necessary and Sufficient Conditions for Fuzzy *a*-Filters and Fuzzy Associative Filters

Checking the proof of Theorem 18 in detail, we know that the proof only applies the properties of fuzzy filters and the conditions in Lemma 17. From this, we can get the following.

**Lemma 19**

Let *μ* be a fuzzy filter of a pseudo-*BCI* algebra *X*. If *μ* satisfies

(C1) for all *x*, *y* ∈ *X*, *μ*(*y*) ≥ *μ*(*x* → (*x*⇝*y*));
(C2) for all *x*, *y* ∈ *X*, *μ*(*y*) ≥ *μ*(*x*⇝(*x* → *y*)),
 then the following statements hold:
(C3) for all *x* ∈ *X*, *μ*(*x* → (*x*⇝1)) = *μ*(*x*⇝(*x* → 1)) = *μ*(1);
(C4) for all *x*, *y* ∈ *X*, *μ*(*x* → *y*) = *μ*(*x*⇝*y*);
(C5) for all *x* ∈ *X*, *μ*(*x*) = *μ*(*x* → 1) = *μ*(*x*⇝1);
(C6) for all *x*, *y* ∈ *X*, *μ*(*x*) ≥ min⁡{*μ*(*y*), *μ*(*x* → *y*)}, *μ*(*x*) ≥ min⁡{*μ*(*y*), *μ*(*x*⇝*y*)}.

**Proof**

It is similar to Theorem 18 (the conditions (FAF1) and (FAF2) are not applied); the proof is omitted.

**Theorem**

Let *μ* be a fuzzy filter of a pseudo-*BCI* algebra *X*. Then *μ* is a fuzzy associative filter of *X* if and only if it satisfies

(C1) for all *x*, *y* ∈ *X*, *μ*(*y*) ≥ *μ*(*x* → (*x*⇝*y*));
(C2) for all *x*, *y* ∈ *X*, *μ*(*y*) ≥ *μ*(*x*⇝(*x* → *y*)).

**Proof**

Assume that *μ* is a fuzzy associative filter of *X*; by Lemma 17, (C1) and (C2) hold.

Conversely, assume that *μ* satisfies conditions (C1) and (C2). For any *x*, *y*, *z* ∈ *X*, by Proposition 2(6), *y* → (*x* → *z*)≤(*x* → *y*)→(*x* → (*x* → *z*)). Using Theorem 9, we get

(28) μ(x⟶(x⟶z)) ≥min⁡{μ(y⟶(x⟶z)),μ(x⟶y)}.

By Lemma 19(C4) and Proposition 2(4), *μ*(*y* → (*x* → *z*)) = *μ*(*y*⇝(*x* → *z*)) = *μ*(*x* → (*y*⇝*z*)).

Thus,

(P1) *μ*(*x* → (*x* → *z*)) ≥ min⁡{*μ*(*x* → (*y*⇝*z*)), *μ*(*x* → *y*)}.

On the other hand, applying Lemma 19(C5), Proposition 2(11), and (12),

(29) μ(x⟶(x⟶z)) =μ((x⟶(x⟶z))⟶1) =μ((x⟶1)⇝((x⟶1)⇝(z⟶1))) =μ((x⟶1)⇝(z⟶((x⟶1)⇝1))) =μ(z⟶((x⟶1)⇝((x⟶1)⇝1))).  

And, by Lemma 19(C6),

(30) μ(z)≥min⁡{μ((x⟶1)⇝((x⟶1)⇝1)),     μ(z⟶((x⟶1)⇝((x⟶1)⇝1)))}.

By Lemma 19(C3) and the above result, we get

(P2) *μ*(*z*) ≥ min⁡{*μ*(1), *μ*(*x* → (*x* → *z*))} = *μ*(*x* → (*x* → *z*)).

Combining (P1) and (P2), we get that *μ*(*z*) ≥ min⁡{*μ*(*x* → (*y*⇝*z*)), *μ*(*x* → *y*)}. That is, (FAF1) holds. Similarly, condition (FAF2) holds. Therefore, by Definition 12, *μ* is a fuzzy associative filter of *X*.

**Theorem (see [13, 15])**

Let *μ* be a fuzzy filter of a pseudo-*BCI* algebra *X*. Then *μ* is a fuzzy *a*-filter of *X* if and only if it satisfies

(a1) for all *x*, *y* ∈ *X*, *μ*(*x* → *y*) ≥ *μ*((*y*⇝1) → *x*);
(a2) for all *x*, *y* ∈ *X*, *μ*(*x*⇝*y*) ≥ *μ*((*y* → 1)⇝*x*).

**Theorem 22**

Let *μ* be a fuzzy filter of a pseudo-*BCI* algebra *X*. Then the following conditions are equivalent:

- *μ* is a fuzzy *a*-filter of *X*;
- *μ* is a fuzzy associative filter of *X*.

**Proof**

(i) ⇒ (ii). Suppose that *μ* is a fuzzy *a*-filter of *X*. For any *x*, *y* ∈ *X*, by Definition 1(1),

(31) x⟶(x⇝1) ≤((x⇝1)⟶(x⇝y))⇝(x⟶(x⇝y)).

Applying Theorem 16(6) and Proposition 7, we get

(32) μ(1)=μ(x⟶(x⇝1))≤μ(((x⇝1)⟶(x⇝y))⇝(x⟶(x⇝y))).

It follows that

(33) $$\begin{array}{c} \mu \left(1\right)=\mu \left(\left(\left(x⇝1\right)⟶\left(x⇝y\right)\right)⇝\left(x⟶\left(x⇝y\right)\right)\right). \end{array}$$

Moreover, by Theorem 16(1),

(34) μ((x⇝1)⟶(x⇝y)) ≥min⁡{μ(x⟶(x⇝y)),      μ(((x⇝1)⟶(x⇝y))      ⇝(x⟶(x⇝y)))}.

Then we get

(35) μ((x⇝1)⟶(x⇝y))≥min⁡⁡{μ(x⟶(x⇝y)),μ(1)}=μ(x⟶(x⇝y)).

Using Theorem 16(2), *μ*((*x*⇝1)⇝(*x*⇝*y*)) = *μ*((*x*⇝1)→(*x*⇝*y*)). Thus,

(36) $$\begin{array}{c} \mu \left(\left(x⇝1\right)⇝\left(x⇝y\right)\right)\geq \mu \left(x⟶\left(x⇝y\right)\right). \end{array}$$

On the other hand, applying Proposition 2(6) and (8), *y* ≤ 1 → *y* ≤ (*x*⇝1)⇝(*x*⇝*y*). From this and Theorem 16(1), we get

(37) μ(y)≥min⁡{μ((x⇝1)⇝(x⇝y)),  μ(y⟶((x⇝1)⇝(x⇝y)))}.

From *y* ≤ (*x*⇝1)⇝(*x*⇝*y*), we have *y* → ((*x*⇝1)⇝(*x*⇝*y*)) = 1; it follows that

(38) μ(y)≥min⁡⁡{μ((x⇝1)⇝(x⇝y)),μ(1)}=μ((x⇝1)⇝(x⇝y)).

Therefore,

(39) $$\begin{array}{c} \mu \left(y\right)\geq \mu \left(\left(x⇝1\right)⇝\left(x⇝y\right)\right)\geq \mu \left(x⟶\left(x⇝y\right)\right). \end{array}$$

This means that (C1) holds. Similarly, *μ*(*y*) ≥ *μ*(*x*⇝(*x* → *y*)). By Theorem 20, *μ* is a fuzzy associative filter of *X*.

(ii) ⇒ (i). Suppose that *μ* is *a* fuzzy associative filter of *X*. For any *x*, *y* ∈ *X*, by Definition 1(1), (*y*⇝1) → *x* ≤ (*x* → *y*)⇝(*y*⇝1). Applying Proposition 7, we get

(40) $$\begin{array}{c} \mu \left(\left(x⟶y\right)⇝\left(y⇝1\right)\right)\geq \mu \left(\left(y⇝1\right)⟶x\right). \end{array}$$

On the other hand, using Theorem 18(12),

(41) μ(x⟶y)≥min⁡{μ((y⇝1)⟶y),  μ((x⟶y)⇝(y⇝1))}.

By Theorem 18(9), *μ*((*y*⇝1) → *y*) = *μ*(1). It follows that

(42) μ(x⟶y)≥min⁡{μ(1),μ((x⟶y)⇝(y⇝1))}=μ((x⟶y)⇝(y⇝1)).

Therefore,

(43) μ(x⟶y)≥μ((x⟶y)⇝(y⇝1))≥μ((y⇝1)⟶x).

Similarly, we can get *μ*(*x*⇝*y*) ≥ *μ*((*y* → 1)⇝*x*). By Theorem 21, *μ* is a fuzzy *a*-filter of *X*.

Now, we discuss the relationship between associative filters and *a*-filters of pseudo-*BCI* algebras. At first, we give the following results (the proofs are omitted).

**Proposition 23**

A nonempty subset *F* of pseudo-*BCI* algebra *X* is a filter (associative filter, *a*-filter) of *X* if and only if the characteristic function *χ*_*F*_ of *F* is a fuzzy filter (fuzzy associative filter, fuzzy *a*-filter) of *X*.

**Proposition 24**

Let *X* be a pseudo-*BCI* algebra. Then a fuzzy set *μ* : *X* → [0,1] is a fuzzy associative filter (fuzzy *a*-filter) of *X* if and only if the level set *μ*_*t*_ = {*x* ∈ *X* | *μ*(*x*) ≥ *t*} is associative filter (*a*-filter) of *X* for all *t* ∈ *Im*⁡(*μ*).

In fact, the above proposition is a consequence of the so-called* Transfer Principle for Fuzzy Sets* in [11].

Combining Propositions 23 and 24, Theorems 8 and 22, we get the following.

**Theorem 25**

Let *F* be a filter of a pseudo-*BCI* algebra *X*. Then the following conditions are equivalent:

- *F* is an *a*-filter of *X*;
- *F* is an associative filter of *X*.

**Remark 26**

In [10], the authors proved Theorem 18, but the proof is wrong (from “Conversely …” to the end of proof). In fact, by Theorem 25, Theorem 4 in [10] is true.

Finally, we discuss the relationship among fuzzy associative filters, fuzzy *a*-filters, and fuzzy *q*-filters in pseudo-*BCI* algebras.

**Lemma 27**

Let *μ* be a fuzzy *p*-filter of a pseudo-*BCI* algebra *X*. Then *μ* satisfies

1. for all *x* ∈ *X*, *μ*(*x*) = *μ*((*x* → 1)⇝1);
2. for all *x* ∈ *X*, *μ*(((*x* → 1)⇝1)⇝*x*) = *μ*(1).

**Proof**

(1) For any *x* ∈ *X*, by Definition 10(FPF1), we have

(44) μ(x)≥min⁡{μ((x→1)⇝(1→1)),μ(1)}=μ((x→1)⇝1).

And, using Definition 1(2) and Proposition 7, *μ*((*x* → 1)⇝1) ≥ *μ*(*x*). It follows that *μ*(*x*) = *μ*((*x* → 1)⇝1).

(2) For any *x* ∈ *X*, by Proposition 2(11), (12), and (9), we have

(45) ((((x⟶1)⇝1)⇝x)⟶1)⇝1 =((((x⟶1)⇝1)⇝x)⟶1)⟶1 =((((x⟶1)⇝1)⇝x)⇝1)⟶1 =((((x⟶1)⇝1)⇝1)⟶(x⇝1))⟶1 =((((x⟶1)⇝1)⟶1)⟶(x⇝1))⟶1 =((x⟶1)⟶(x⇝1))⟶1 =((x⟶1)⟶(x⟶1))⟶1 =1⟶1=1.

From this and (1), we get

(46) μ(((x⟶1)⇝1)⇝x) =μ(((((x⟶1)⇝1)⇝x)⟶1)⇝1)=μ(1).

This means that (2) holds.

**Theorem 28**

Let *μ* be a fuzzy filter of a pseudo-*BCI* algebra *X*. Then the following conditions are equivalent:

1. *μ* is a fuzzy *a*-filter of *X*;
2. *μ* is both a fuzzy *p*-filter and a fuzzy *q*-filter of *X*.

**Proof**

Assume that *μ* is a fuzzy *a*-filter of *X*. It is easy to prove that *μ* is both a fuzzy *p*-filter and a fuzzy *q*-filter of *X*.

Conversely, let *μ* be both a fuzzy *p*-filter and fuzzy *q*-filter of *X*. For any *x*, *y* ∈ *X*, by Definition 13(FqF1), we have

(47) μ(x⟶y) ≥min⁡{μ((x⇝((y⇝1)⟶x))⟶y),    μ((y⇝1)⟶x)}.

And, by Proposition 2(4),

(48) (x⇝((y⇝1)⟶x))⟶y =((y⇝1)⟶(x⇝x))⟶y =((y⇝1)⟶1)⟶y.

From this and Lemma 27(2),

(49) μ((x⇝((y⇝1)⟶x))⟶y) =μ(((y⇝1)⟶1)⟶y)=μ(1).

Therefore,

(50) μ(x⟶y) ≥min⁡{μ(1),μ((y⇝1)⟶x)}=μ((y⇝1)⟶x).

This means that Theorem 21(a1) holds. Similarly, we can prove (a2). By Theorem 21, we know that *μ* is a fuzzy *a*-filter of *X*.

## References

[1] Gabbay DM, Hogger CJ, Robinson JA (1994). *Handbook of Logic in Artificial Intelligence and Logic Programming*.

[2] Iséki K (1966). An algebra related with a propositional calculus. *Proceedings of the Japan Academy*.

[3] Zhang X (2013). BCC-algebras and residuated partially-ordered groupoid. *Mathematica Slovaca*.

[4] Ma X, Zhan J, Jun YB (2012). New types of fuzzy ideals of BCI-algebras. *Neural Computing and Applications*.

[5] Zhang X-H, Li WH (2006). On pseudo-BL algebras and BCC-algebras. *Soft Computing*.

[6] Dudek WA, Jun YB (2008). Pseudo-BCI algebras. *East Asian Mathematical Journal*.

[7] Georgescu G, Iorgulescu A, Calude CS, Dinneen MJ, Sburlan S (2001). Pseudo-BCK algebras: an extension of BCK algebras. *Combinatorics, Computability and Logic*.

[8] Zhang X, Lu Y, Mao X T-type pseudo-BCI algebras and T-type pseudo-BCI filters.

[9] Jun YB, Kim HS, Neggers J (2006). On pseudo-BCI ideals of pseudo-BCT algebras. *Matematicki Vesnik*.

[10] Lee KJ, Park CH (2009). Some ideals of pseudo BCI-algebras. *Journal of Applied Mathematics & Informatics*.

[11] Kondo M, Dudek WA (2005). On the transfer principle in fuzzy theory. *Mathware & Soft Computing*.

[12] Wang W, Xin X-L (2011). On fuzzy filters of pseudo BL-algebras. *Fuzzy Sets and Systems*.

[13] Gilani A, Waphare BN (2007). On pseudo *a*-ideal of pseudo-BCI algebras. *Scientia Magna*.

[14] Jun YB, Song SZ (2003). Fuzzy pseudo-ideals of pseudo-BCK algebras. *Journal of Applied Mathematics and Computing*.

[15] Lee KJ (2009). Fuzzy ideals of pseudo BCI-algebras. *Journal of Applied Mathematics & Informatics*.

